# Multicenter, Randomized, Phase III Trial of Neoadjuvant Chemoradiation With Capecitabine and Irinotecan Guided by *UGT1A1* Status in Patients With Locally Advanced Rectal Cancer

**DOI:** 10.1200/JCO.20.01932

**Published:** 2020-10-29

**Authors:** Ji Zhu, Anwen Liu, Xinchen Sun, Luying Liu, Yaqun Zhu, Tao Zhang, Jianhui Jia, Shisheng Tan, Junxin Wu, Xin Wang, Juying Zhou, Jialin Yang, Chen Zhang, Hongyan Zhang, Yuanyuan Zhao, Gang Cai, Wei Zhang, Fan Xia, Juefeng Wan, Hui Zhang, Lijun Shen, SanJun Cai, Zhen Zhang

**Affiliations:** ^1^ Department of Radiation Oncology, Fudan University Shanghai Cancer Center, Shanghai, China; ^2^ Department of Oncology, Second Affiliated Hospital of Nanchang University, Nanchang, China; ^3^ Department of Radiation Oncology, First Affiliated Hospital of Nanjing Medical University, Nanjing, China; ^4^ Department of Radiation Oncology, Cancer Hospital of the University of Chinese Academy of Sciences & Zhejiang Cancer Hospital, Hangzhou, China; ^5^ Department of Radiation Oncology, Second Affiliated Hospital of Soochow University, Suzhou, China; ^6^ Department of Oncology, The First Affiliated Hospital of Chongqing Medical University, Chongqing, China; ^7^ Department of Radiotherapy, Liaoning Cancer Hospital & Institute, China Medical University Cancer Hospital, Shenyang, China; ^8^ Department of Oncology, Guizhou Provincial People’s Hospital, Guiyang, China; ^9^ Department of Radiation Oncology, Fujian Provincial Cancer Hospital, Fuzhou, China; ^10^ Department of Abdominal Oncology, West China Hospital Sichuan University, Chengdu, China; ^11^ Department of Radiation Oncology, First Affiliated Hospital of Soochow University, Suzhou, China; ^12^ Department of Radiation Oncology, Sichuan Cancer Hospital& Institute, Chengdu, China; ^13^ Department of Radiation Oncology, HWA MEI Hospital, University of Chinese Academy of Science, Ningbo, China; ^14^ Department of Radiation Oncology, The First Affiliated Hospital of USTC, Division of Life Sciences and Medicine, University of Science and Technology of China, Hefei, Anhui, China; ^15^ Department of Radiation Oncology, The Affiliated Hospital of Qingdao University, Qingdao, China; ^16^ Department of Radiation Oncology, Ruijin Hospital Shanghai Jiaotong University School of Medicine, Shanghai, China; ^17^ Department of Biostatistics, School of Public Health, Fudan University, Shanghai, China; ^18^ Department of Colorectal Cancer, Fudan University Shanghai Cancer Center, Shanghai, China; ^19^ Department of Oncology, Shanghai Medical College, Fudan University, Shanghai, China

## Abstract

Differentiating the irinotecan dose on the basis of the uridine diphosphate glucuronosyltransferase 1A1 (*UGT1A1*) genotype improves the pathologic complete response (pCR) rate. In this study, we further investigated preoperative irinotecan combined with capecitabine-based chemoradiotherapy for locally advanced rectal cancer.

**PATIENTS AND METHODS:**

We conducted this randomized, open-label, multicenter, phase III trial in China. Eligible patients with clinical T3-4 and/or N+ rectal adenocarcinoma, *UGT1A1* genotype *1*1 or *1*28 were randomly allocated to the control group: pelvic radiation of 50 Gy/25 fractions with concurrent capecitabine, followed by oxaliplatin and capecitabine; or the experimental group: radiation with capecitabine combined with weekly irinotecan 80 mg/m^2^ for patients with UGT1A1*1*1 or 65 mg/m^2^ for patients with UGT1A1*1*28, followed by irinotecan and capecitabine. The primary end point was pCR. This trial was registered with ClinicalTrials.gov (ClinicalTrials.gov identifier: NCT02605265).

**RESULTS:**

Of the 360 patients initially enrolled, 356 were evaluated as the modified intention-to-treat population (n = 178 in both groups). Surgery was performed in 87% and 88% of patients in the control and experimental groups, respectively. The pCR rates were 15% (n = 27 of 178) and 30% (n = 53 of 178) in the control and experimental groups (risk ratio, 1.96; 95% CI, 1.30 to 2.97; *P* = .001). Four and 6 patients achieved complete clinical response in the control and experimental groups, respectively. Grade 3-4 toxicities were recorded in 11 (6%) and 68 (38%) patients in the control and experimental groups, respectively (*P* < .001). The commonest grade 3-4 toxicities were leukopenia, neutropenia, and diarrhea. The overall surgical complication rate was not significantly different between the two groups (11% *v* 15%; *P* < .001).

**CONCLUSION:**

Adding irinotecan guided by *UGT1A1* genotype to capecitabine-based neoadjuvant chemoradiotherapy significantly increased complete tumor response in Chinese patients.

## INTRODUCTION

Preoperative chemoradiotherapy (CRT) followed by surgery is the standard treatment of locally advanced rectal cancer.^1,2^ However, the pathologic complete response (pCR) rate is only 10% to 15%, and the distant metastasis rate is > 30%. Adding a second drug during neoadjuvant treatment may yield a better tumor response and reduce the risk of metastasis.^3^ Oxaliplatin showed a good clinical outcome in early exploratory studies. However, phase III trials did not confirm that addition of oxaliplatin to neoadjuvant CRT improved the pCR rate or long-term survival; instead, it caused more treatment-related adverse events.^4-8^ Therefore, whether adding a second drug to neoadjuvant CRT can improve the clinical outcome remains controversial.

**CONTEXT**

**Key Objective**
Irinotecan has been investigated for its efficacy when combined with neoadjuvant chemoradiotherapy (CRT), but there has been concern about its poor tolerability. The *UGT1A1* genotype has become the key determinant of irinotecan dosing. In this trial, we assessed the benefit of adding irinotecan to the treatment of patients undergoing neoadjuvant chemoradiotherapy. To our knowledge, this is the first phase III trial to use *UGT1A1* genotype to guide irinotecan dose when used in combination with capecitabine-based neoadjuvant CRT.
**Knowledge Generated**
The results showed that additional irinotecan guided by *UGT1A1* genotype can increase the pCR rate from 15% to 30% compared with capecitabine-based neoadjuvant chemoradiotherapy. More toxicities were induced by irinotecan, particularly leukopenia, neutropenia, and diarrhea.
**Relevance**
Under the guidance of *UGT1A1* genotype, an increased dose of irinotecan added to standard CRT might become an improved strategy to achieve a better tumor regression in patients with locally advanced rectal cancer.

The efficacy of irinotecan as combination neoadjuvant CRT has been investigated in studies with small sample sizes.^9-13^ However, there is concern about its poor tolerability in Western populations and especially its propensity to cause neutropenia and diarrhea. The maximum tolerated dose (MTD) of irinotecan has been determined to be only 40-60 mg/m^2^ per week when used concurrently with 5-fluorouracil–based CRT.^14,15^ In the RTOG 0247 study, which explored irinotecan and oxaliplatin in combination with capecitabine-based neoadjuvant CRT, the doses of irinotecan and capecitabine needed to be reduced significantly, which might explain the worse pCR rate with irinotecan (10% in the irinotecan group *v* 21% in the oxaliplatin group) reported in that study.^16^ However, the irinotecan group demonstrated higher overall and disease-free survival rates than did the oxaliplatin group.^17^

More recently, uridine diphosphate glucuronosyltransferase 1A1 (*UGT1A1*) activity has become the key determinant of whether the irinotecan dose can be increased.^18-20^ Irinotecan is converted to SN-38, which is subsequently inactivated by *UGT1A1* and excreted via the bile.^21^ There is an important association between the *UGT1A1* genotype and the SN-38 inactivation rate, which influences the likelihood of toxicity. Several dose-escalation studies have found that the MTD of irinotecan decreased with an increasing number of defective *UGT1A1* alleles,^22-25^ confirming that the irinotecan dose can be guided by the *UGT1A1* genotype.

We hypothesized that the irinotecan dose administered to patients undergoing neoadjuvant CRT could be guided by the *UGT1A1* genotype. In our previous dose-escalation and expansion studies, 80 mg/m^2^ and 65 mg/m^2^ were identified as the weekly MTDs of irinotecan for patients with the UGT1A1*1*1 and *1*28 genotypes, respectively, when administered in combination with capecitabine-based CRT.^26^ The aim of the current study was to assess the benefit of adding irinotecan to treatment of patients undergoing neoadjuvant CRT.

## PATIENTS AND METHODS

### Study Design and Participants

In this multicenter, randomized, prospective, open-label phase III clinical trial, we compared the therapeutic benefit of capecitabine-based neoadjuvant CRT with that of irinotecan plus capecitabine-based CRT in patients with locally advanced rectal cancer who were treated at 17 radiation oncology centers in China. The study protocol was approved by the central ethics committee of Fudan University Shanghai Cancer Center (Shanghai, China) and the institutional review boards of all participating institutions. All patients provided written informed consent before participation.

Patients were eligible if they were aged 18-75 years and had histopathologically confirmed rectal adenocarcinoma located ≤ 10 cm above the anal verge and clinical stage T3-4 and/or N+ disease on pelvic magnetic resonance images. Conventional chest and abdominal computed tomography (CT) scans were used to confirm the absence of distant metastases. Other inclusion criteria were a Karnofsky performance status score ≥ 70, a *UGT1A1* genotype of *1*1 or *1*28, and adequate bone marrow function (defined as a hemoglobin level ≥ 9 g/dL, neutrophil count ≥ 1,500/μL, and platelet count ≥ 100,000/μL), liver function (total bilirubin level < 1.5 times the upper limit of normal; albumin level > 30 g/L; and aspartate aminotransferase, alanine aminotransferase, and alkaline phosphatase levels < 2.5 times the upper limit of normal), and kidney function (creatinine concentration below the upper limit of normal).

The exclusion criteria were a history of malignancy, with the exception of adequately treated basal cell carcinoma of the skin or cervical carcinoma in situ; previous chemotherapy or pelvic radiotherapy; pregnancy or lactation; known dihydropyrimidine dehydrogenase deficiency; or a serious illness, such as unstable angina or myocardial infarction, within the previous 12 months.

### *UGT1A1* Genotyping

All patients underwent *UGT1A1* genotyping. Before treatment, 2 mL of venous blood was collected, and blood samples were collected using DNA extraction kits (QIAamp DNA Blood Midi Kit; QIAGEN, Venlo, the Netherlands). Polymerase chain reaction assay was performed in a 25-µL reaction with 2.5 µL of 15 mM Mg^2+^, 2 µL of 2.5 mM deoxyribonucleotide triphosphates, 5 U Taq, and 30 ng of DNA. The following primers were used: forward, 5′-TCC​CTG​CTA​CCT​TTG​TGG​AC-3′ and reverse, 5′-AGC​AGG​CCC​AGG​ACA​AGT-3′. The reaction was run for 40 cycles at 94°C for 15 seconds, 60°C for 25 seconds, and 72°C for 30 seconds. Genotypes were assigned on the basis of the number of thymine-adenine repeats in each allele.

### Randomization and Masking

Eligible patients were randomly allocated to receive radiotherapy with concurrent capecitabine (CapRT) or concurrent capecitabine and irinotecan (CapIriRT). The patients were assigned centrally through a web interface (varied permuted block design with a block size 2-6) hosted by the Fudan University Biostatistics Central Office (Shanghai, China). The patients were stratified by clinical T stage (cT3 *v* cT4), tumor distance from the anal verge (≤ 5 cm *v* > 5 cm), and *UGT1A1* genotype (*1*1 *v* *1*28).

### Treatment Procedure

The control group (CapRT) received pelvic radiation at a dose of 50 Gy/25 fractions, delivered with a 6-10-MV photon beam via intensity-modulated radiation therapy with concurrent capecitabine 825 mg/m^2^ twice daily 5 d/wk, followed by a cycle of capcecitabine plus oxaliplatin (XELOX) 2 weeks after the end of CRT (oxaliplatin 130 mg/m^2^ on day 1 and capecitabine 1,000 mg/m^2^ twice daily on days 1-14). The experimental group (CapIriRT) received pelvic radiation at a dose of 50 Gy/25 fractions with capecitabine 625 mg/m^2^ twice daily 5 d/wk and weekly irinotecan, followed by a cycle of capecitabine plus irinotecan (XELIRI) 2 weeks after completion of CRT (irinotecan 200 mg/m^2^ on day 1 and capecitabine 1,000 mg/m^2^ twice daily on days 1-14). Irinotecan was administered at a weekly dose of 80 mg/m^2^ among patients with the UGT1A1*1*1 genotype and 65 mg/m^2^ among those with the UGT1A1*1*28 genotype. Details of the irradiation techniques and treatment volumes are provided in the Data Supplement.

Surgery was scheduled for 8 weeks after completion of CRT. Total mesorectal excision was mandatory, whereas the surgical approach (anterior resection or abdominal-perineal resection [APR]) and whether a temporary colostomy was used were at the discretion of the surgeon. Other types of surgery (Hartmann’s procedure, intersphincteric resection, and transanal local excision) were permissible at the surgeon’s discretion. Five cycles of adjuvant XELOX chemotherapy were administered regardless of the pathologic result.

### Pathology Procedures

Resected specimens were processed and examined as previously described.^27^ Pathologists were blinded to each patient’s treatment plan and evaluated surgical specimens independently. The extent of residual tumor was classified according to the eighth edition of the International Union Against Cancer’s TNM staging system. All resected lymph nodes were examined according to standard procedures. If there were < 12 lymph nodes, two pathologists performed the examination to ensure reliable results. pCR was defined as an absence of tumor cells in the surgical specimens from the primary tumor and the regional lymph nodes (ypT0N0). Tumor regression grade was evaluated according to the criteria devised by Edge et al.^28^

### Data Management and Statistical Analysis

This study was primarily designed to detect an increase in the pCR rate from 12% in the CapRT group to 25% in the CapIriRT group, and the pCR rate in the intention-to-treat (ITT) population was the primary end point. According to our calculation, 360 eligible patients would need to be recruited to detect this difference with an α of 0.05 (two-tailed) and a β of 0.15. However, some patients refused to undergo surgery because they obtained a complete response to neoadjuvant therapy; therefore, two sensitivity analyses were planned to confirm stable results, including the pCR rate in the surgical population and the overall CR rate (pCR plus clinical complete response [cCR]) in the ITT population.

For patients who had a good tumor response and refused APR, a watch-and-wait policy was adopted. These patients received six cycles of consolidation chemotherapy and were monitored closely with digital examination and endoscopy every 1-2 months and chest and abdominal CT and pelvic magnetic resonance imaging (MRI) every 3 months. The cCR was defined as the absence of palpable tumor on digital examination, the absence of residual tumor on pelvic MRI or endoscopy, and a sustained absence of residual tumor for at least 12 months after CRT.

The secondary end points included toxicities, quality of life, tumor regression grade, sphincter preservation, surgical complications, local control, disease-free survival (DFS), and overall survival (OS). Preoperative acute toxicity and surgical complications were recorded according to the National Cancer Institute Common Toxicity Criteria, version 4.0. Survival time was calculated from the date of randomization to the date of event or the last follow-up. Events were defined as local failure for local control, tumor recurrence or death from any cause for DFS, and death from any cause for OS. Categorical variables are presented as frequencies and continuous variables as means and standard deviations if normally distributed or medians if not normally distributed. Categorical variables were compared between the two groups using the χ^2^ test or Fisher exact test with the log-rank test for survival data. Analysis items for which *P* < .05 were considered statistically significant.

## RESULTS

**FIG 1.**
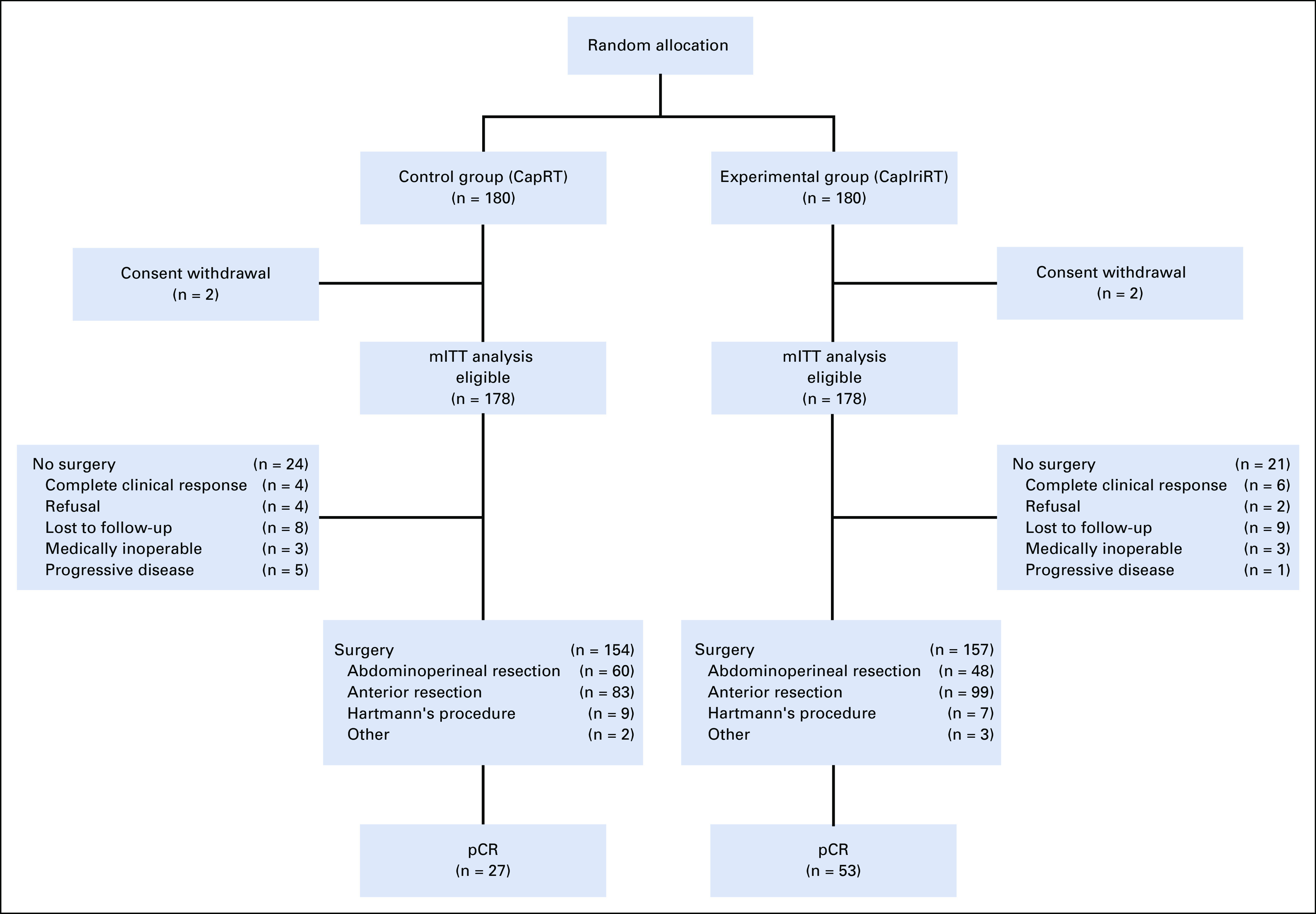
CONSORT diagram. CapRT, radiotherapy at a dose of 50 Gy with concurrent capecitabine; CapIriRT, radiotherapy at a dose of 50 Gy with concurrent capecitabine and irinotecan; mITT, modified intention-to-treat; pCR, pathologic complete response.

From November 2015 to December 2017, 360 patients were recruited at 17 centers in China; nine patients with a *28*28 genotype were excluded. After randomization, we excluded four patients who withdrew consent to participate before receiving any treatment. As a post hoc decision, the remaining 356 patients constituted the modified ITT (mITT) population, replacing the ITT population for additional analysis. We allocated 178 patients to each treatment group (Fig 1). The patients’ baseline demographic and clinical characteristics were well balanced (Table 1).

**TABLE 1.**
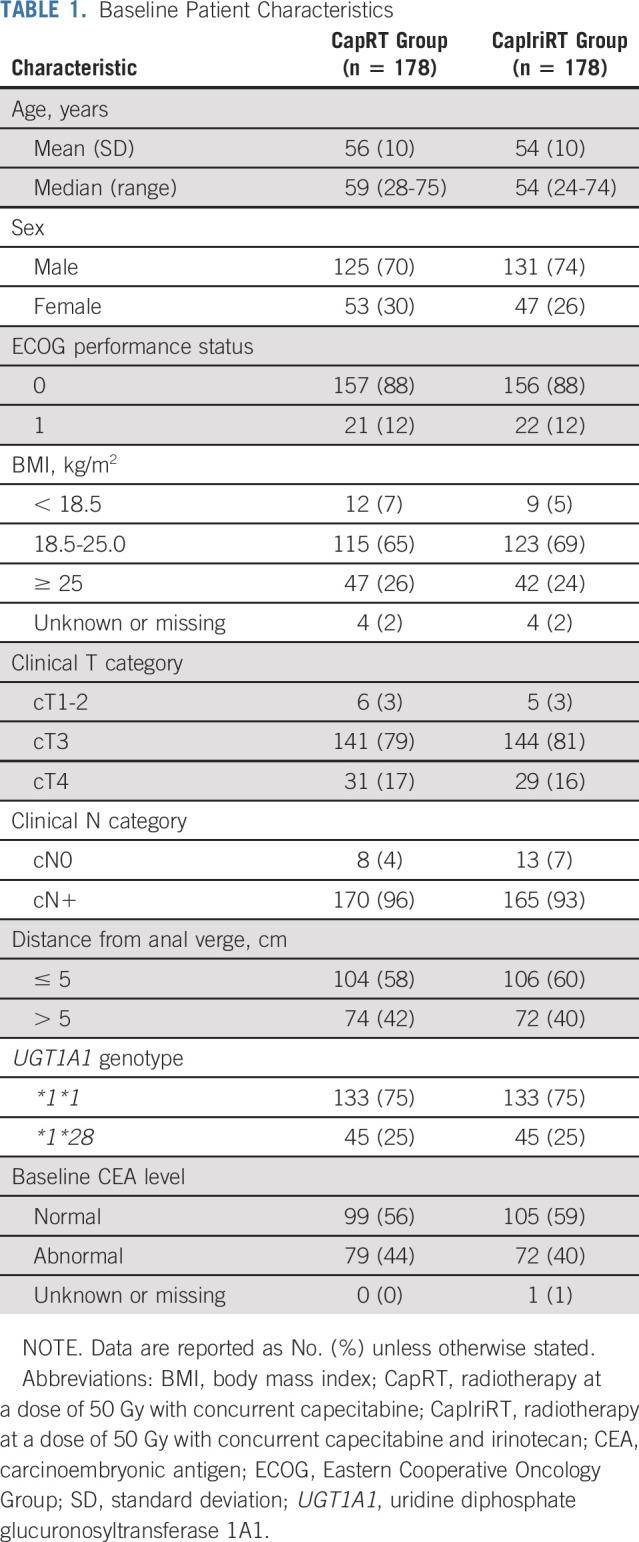
Baseline Patient Characteristics

All patients (100%) in the CapRT group received ≥ 90% of the full dose of radiotherapy and capecitabine. In the CapIriRT group, 175 patients (98%) received ≥ 90% of the full dose of radiotherapy and capecitabine, and 125 (70%) received at least four cycles of weekly irinotecan (Table 2).

**TABLE 2.**
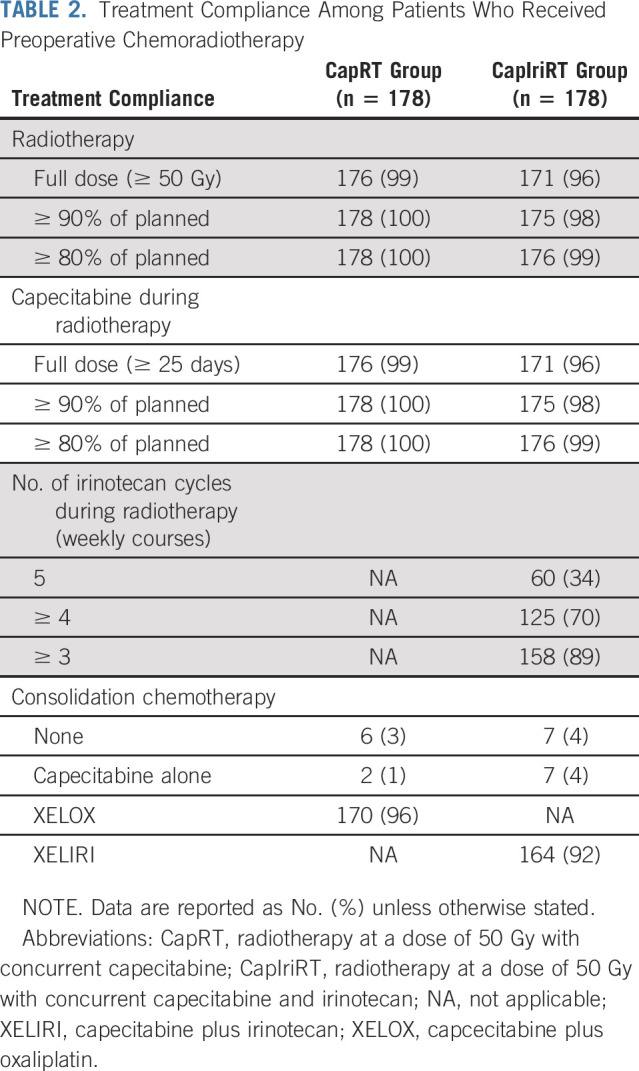
Treatment Compliance Among Patients Who Received Preoperative Chemoradiotherapy

After neoadjuvant CRT completion, six patients (3%) in the CapRT group and seven (4%) in the CapIriRT group did not receive any consolidation chemotherapy. In total, 170 patients (96%) in the CapRT group received XELOX and 164 (92%) in the CapIriRT group received XELIRI. Another two patients (1%) in the CapRT group and seven (4%) in the CapIriRT group received one cycle of capecitabine alone between the end of CRT and surgery (Table 2).

Grade 3-4 toxic effects were recorded among 11 patients (6%) in the CapRT group and 68 (38%) in the CapIriRT group (*P* < .001; Table 3). The most common grade 3-4 toxicities during CRT in the CapRT group versus the CapIriRT group, respectively, were leukopenia (3% *v* 25%), neutropenia (2% *v* 20%), and diarrhea (2% *v* 13%).

**TABLE 3.**
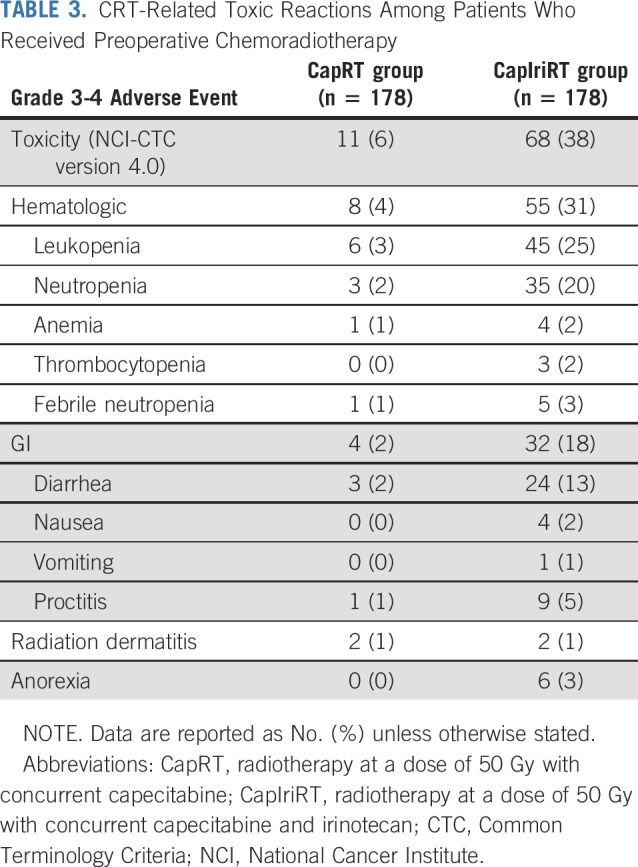
CRT-Related Toxic Reactions Among Patients Who Received Preoperative Chemoradiotherapy

A total of 154 patients (87%) in the CapRT group and 157 (88%) in the CapIriRT group underwent surgery. The median intervals between the end of CRT and surgery were 61 days (range, 45-104 days) in the CapRT group and 62 days (range, 44-156 days) in the CapIriRT group. Sixty patients (39%) in the CapRT group and 48 (31%) in the CapIriRT group underwent APR (*P* = .120; Table 4). The two groups had similar proportions of patients with postoperative complications of grade 3 or worse (11% *v* 15%; *P* = .268). No patient died within 60 days of surgery.

**TABLE 4.**
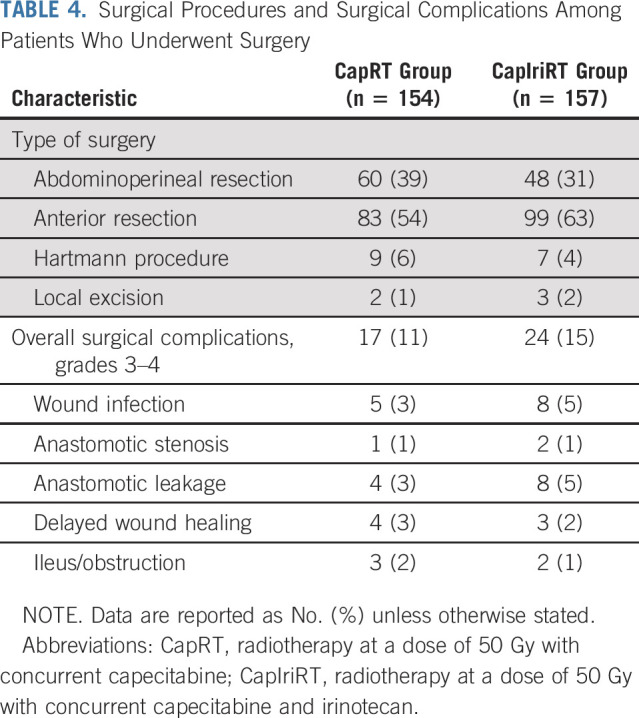
Surgical Procedures and Surgical Complications Among Patients Who Underwent Surgery

Complete resection was achieved in 148 patients (96%) in the CapRT group and 153 (97%) in the CapIriRT group, with circumferential resection margins of ≤ 1 mm in seven (5%) and three (2%) patients, respectively (Table 5). pCR was achieved in 27 patients (18%) in the CapRT group and 53 (34%) in the CapIriRT group (risk ratio, 1.93; 95% confidence interval [CI], 1.28 to 2.89; *P* = .001) in the surgical population. Negative nodes were reported in 112 patients (73%) in the CapRT group and 116 (74%) in the CapIriRT group (Table 5). The pCR rates in the mITT population were 15% (n = 27 of 178) in the CapRT group and 30% (n = 53 of 178) in the CapIriRT group (risk ratio, 1.96; 95% CI, 1.30 to 2.97; *P* = .001).

**TABLE 5.**
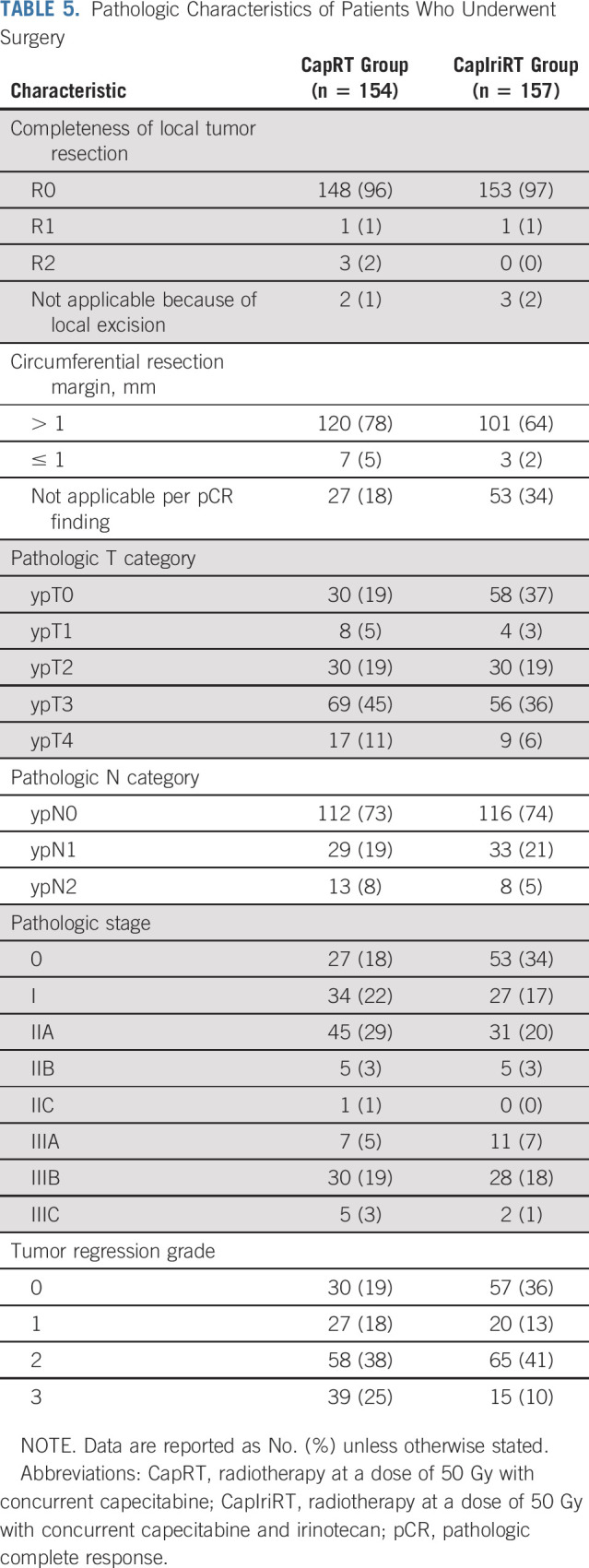
Pathologic Characteristics of Patients Who Underwent Surgery

In the CapRT group, four patients opted to undergo a watch-and-wait approach after achieving cCR, and 20 did not proceed to surgery for various reasons, including refusal, loss to follow-up, being medically inoperable, and progressive disease; the respective numbers in the CapIriRT group were 6 and 15. The two sensitivity analyses showed similar results for the primary end point (Data Supplement).

## DISCUSSION

To our knowledge, this is the first phase III trial to evaluate the use of the *UGT1A1* genotype to guide the irinotecan dose when used in combination with capecitabine-based neoadjuvant CRT in patients with rectal cancer. The primary end point was reached in that the pCR rate increased from 15% in the CapRT group to 30% in the CapIriRT group. However, the addition of irinotecan was also associated with a significant increase in the frequency of grade 3-4 toxicities (38%), particularly leukopenia (25%), neutropenia (20%), and diarrhea (13%), although rates of sphincter preservation and surgical complications remained similar. The local control, DFS, and OS data reflecting the long-term prognosis are not yet mature; we plan to report survival outcomes separately in approximately 3 years.

Irinotecan inhibits topoisomerase I and is an effective chemotherapeutic agent for colorectal cancer. Preclinical studies have shown that radiation kills tumor cells in the G2 phase through M phase but spares cells in the S phase, which are targeted by irinotecan.^29^ Therefore, irinotecan should have a favorable synergistic effect when used in combination with radiotherapy. However, the addition of irinotecan to neoadjuvant CRT did not achieve good tumor regression in previous phase I/II trials (Data Supplement), likely owing to inadequate doses. Klautke et al^30^ reported that pCR was achieved in 16%-35% of patients who received a total irinotecan dose of 240 mg/m^2^ but not in patients who received a total dose of 200 mg/m^2^. Therefore, we hypothesized that irinotecan could be an ideal radiosensitizer only at a sufficient dose. However, because of serious irinotecan-induced toxicities, it has been difficult to verify this hypothesis in clinical trials.

Understanding the relationship between the *UGT1A1* genotype and irinotecan toxicities has renewed interest in therapeutic approaches to rectal cancer. Several trials have reported significant differences in the MTD of irinotecan in patients with *UGT1A1* variants.^20-25,31^ Unfortunately, few studies with large sample sizes have been conducted, possibly because of concerns of toxicity. However, the prevalence of *UGT1A1* variants differs between White and Asian populations. The *1*1 and *1*28 genotypes are observed in 46% and 39% of White patients, respectively,^20^ and 80% and 16% of Asian patients, respectively.^32^ Therefore, the irinotecan dose recommended for White patients may be unsuitable for Asian patients. We conducted a pilot study of *UGT1A1*-guided irinotecan dosing for neoadjuvant CRT. The MTD of irinotecan went up to 80 mg/m^2^ per week in patients with the *1*1 genotype and 65 mg/m^2^ per week in patients with the *1*28 genotype. These doses were significantly higher than those in previous studies and may have contributed to the increased pCR rates.^26,33^ On the basis of those results, we conducted the present phase III trial to assess whether an increased dose of concurrent irinotecan would be beneficial.

Conventionally, radical surgery is scheduled 6-10 weeks after the end of long-course CRT, without any consolidation therapy during the interval between CRT and surgery. Some studies showed that adding consolidation chemotherapy between the end of CRT and surgery could improve clinical outcomes.^34^ However, it is unclear how many cycles of consolidation chemotherapy are optimal. We decided to add one cycle of consolidation chemotherapy without delaying surgery. This strategy has demonstrated efficiency and safety in previous trials.^35,36^ We administered one cycle of XELOX in the control group and one cycle of XELIRI in the experimental group to maintain consistency with the drug used in concurrent CRT. Although XELIRI might be challenged for its tolerance, its good efficacy and acceptable toxicity profiles in Eastern Asian populations have been proven.^37^

This phase III trial evaluating irinotecan in neoadjuvant CRT for patients with rectal cancer under the guidance of the *UGT1A1* genotype has some limitations. First, only the UGT1A1*28 allele was used to guide the irinotecan dose. Other biomarkers, such as the UGT1A1*6 allele, that have also shown a correlation with irinotecan-induced toxicities were not considered. Particularly, the UGT1A1*6 allele shows a compensation for the reduced frequency of the UGT1A1*28 allele in Asians.^38^ Combining the UGT1A1*28 and UGT1A1*6 alleles in our studies and clinical practice warrants more attention. Second, our surgical resection rate of approximately 87% was lower than that in previous phase III trials for oxaliplatin, mainly because some patients with a lower tumor burden refused APR regardless of whether cCR was achieved. During at least 12 months of follow-up, 10 patients were deemed to have achieved cCR. Third, patients in the experimental group were exposed to oxaliplatin, irinotecan, and fluorouracil during the perioperative period. This might complicate treatment options in the event of disease recurrence. However, the RTOG0247 study found a long-term survival benefit from early exposure to three chemotherapy agents.^17^ If our trial demonstrates a longer OS, it is worth re-evaluating the sequence of these three drugs for locally advanced rectal cancer.

The ongoing phase III ARISTOTLE trial being conducted in the United Kingdom was also designed to determine the benefit of irinotecan in neoadjuvant CRT. The main difference between ARISTOTLE and our trial is that patients in ARISTOTLE receive a fixed irinotecan dose of 60 mg/m^2^ per week for four cycles without *UGT1A1* genotype–based guidance. At the 2020 ASCO annual meeting, it was reported that patients in the irinotecan group of ARISTOTLE did not reach a higher pCR rate. We believe the main reason for this was that the irinotecan dose was insufficient. However, as mentioned, there are differences in the genotype prevalence and irinotecan tolerance between White and Asian populations. We need to fully consider such differences when assessing the generalizability of our results.

In conclusion, under the guidance of the *UGT1A1* genotype, an increased irinotecan dose in combination with CRT significantly improved the clinical response rate with acceptable toxicities in Chinese patients with locally advanced rectal cancer.

## Data Availability

Individual participant data and a data dictionary defining each field will be available after publication of long-term results, in compliance with relevant national laws and regulations, via leoon.zhu@gmail.com.

## References

[1] Sauer R Becker H Hohenberger W, et al: Preoperative versus postoperative chemoradiotherapy for rectal cancer. N Engl J Med 351:1731-1740, 2004 15496622 10.1056/NEJMoa040694

[2] Sauer R Liersch T Merkel S, et al: Preoperative versus postoperative chemoradiotherapy for locally advanced rectal cancer: Results of the German CAO/ARO/AIO-94 randomized phase III trial after a median follow-up of 11 years. J Clin Oncol 30:1926-1933, 2012 22529255 10.1200/JCO.2011.40.1836

[3] Hartley A Ho KF McConkey C, et al: Pathological complete response following pre-operative chemoradiotherapy in rectal cancer: Analysis of phase II/III trials. Br J Radiol 78:934-938, 2005 16177017 10.1259/bjr/86650067

[4] Deng Y Chi P Lan P, et al: Modified FOLFOX6 with or without radiation versus fluorouracil and leucovorin with radiation in neoadjuvant treatment of locally advanced rectal cancer: Initial results of the Chinese FOWARC multicenter, open-label, randomized three-arm phase III trial. J Clin Oncol 34:3300-3307, 2016 27480145 10.1200/JCO.2016.66.6198

[5] Rödel C Graeven U Fietkau R, et al: Oxaliplatin added to fluorouracil-based preoperative chemoradiotherapy and postoperative chemotherapy of locally advanced rectal cancer (the German CAO/ARO/AIO-04 study): Final results of the multicentre, open-label, randomised, phase 3 trial. Lancet Oncol 16:979-989, 2015 26189067 10.1016/S1470-2045(15)00159-X

[6] Aschele C Cionini L Lonardi S, et al: Primary tumor response to preoperative chemoradiation with or without oxaliplatin in locally advanced rectal cancer: Pathologic results of the STAR-01 randomized phase III trial. J Clin Oncol 29:2773-2780, 2011 21606427 10.1200/JCO.2010.34.4911

[7] Gérard JP Azria D Gourgou-Bourgade S, et al: Clinical outcome of the ACCORD 12/0405 PRODIGE 2 randomized trial in rectal cancer. J Clin Oncol 30:4558-4565, 2012 23109696 10.1200/JCO.2012.42.8771

[8] O’Connell MJ Colangelo LH Beart RW, et al: Capecitabine and oxaliplatin in the preoperative multimodality treatment of rectal cancer: Surgical end points from National Surgical Adjuvant Breast and Bowel Project trial R-04. J Clin Oncol 32:1927-1934, 2014 24799484 10.1200/JCO.2013.53.7753 PMC4050205

[9] Mohiuddin M Winter K Mitchell E, et al: Randomized phase II study of neoadjuvant combined-modality chemoradiation for distal rectal cancer: Radiation Therapy Oncology Group Trial 0012. J Clin Oncol 24:650-655, 2006 16446336 10.1200/JCO.2005.03.6095

[10] Navarro M Dotor E Rivera F, et al: A phase II study of preoperative radiotherapy and concomitant weekly irinotecan in combination with protracted venous infusion 5-fluorouracil, for resectable locally advanced rectal cancer. Int J Radiat Oncol Biol Phys 66:201-205, 2006 16814947 10.1016/j.ijrobp.2006.04.007

[11] Willeke F Horisberger K Kraus-Tiefenbacher U, et al: A phase II study of capecitabine and irinotecan in combination with concurrent pelvic radiotherapy (CapIri-RT) as neoadjuvant treatment of locally advanced rectal cancer. Br J Cancer 96:912-917, 2007 17325705 10.1038/sj.bjc.6603645 PMC2360100

[12] Gollins SW Myint S Susnerwala S, et al: Preoperative downstaging chemoradiation with concurrent irinotecan and capecitabine in MRI-defined locally advanced rectal cancer: A phase I trial (NWCOG-2). Br J Cancer 101:924-934, 2009 19690550 10.1038/sj.bjc.6605258 PMC2743353

[13] Hong YS Kim DY Lim SB, et al: Preoperative chemoradiation with irinotecan and capecitabine in patients with locally advanced resectable rectal cancer: Long-term results of a Phase II study. Int J Radiat Oncol Biol Phys 79:1171-1178, 2011 20605355 10.1016/j.ijrobp.2009.12.073

[14] Hofheinz RD von Gerstenberg-Helldorf B Wenz F, et al: Phase I trial of capecitabine and weekly irinotecan in combination with radiotherapy for neoadjuvant therapy of rectal cancer. J Clin Oncol 23:1350-1357, 2005 15684318 10.1200/JCO.2005.04.171

[15] Gollins S Sun Myint A Haylock B, et al: Preoperative chemoradiotherapy using concurrent capecitabine and irinotecan in magnetic resonance imaging-defined locally advanced rectal cancer: Impact on long-term clinical outcomes. J Clin Oncol 29:1042-1049, 2011 21263095 10.1200/JCO.2010.29.7697

[16] Wong SJ Winter K Meropol NJ, et al: Radiation Therapy Oncology Group 0247: A randomized phase II study of neoadjuvant capecitabine and irinotecan or capecitabine and oxaliplatin with concurrent radiotherapy for patients with locally advanced rectal cancer. Int J Radiat Oncol Biol Phys 82:1367-1375, 2012 21775070 10.1016/j.ijrobp.2011.05.027 PMC3208721

[17] Wong SJ Moughan J Meropol NJ, et al: Efficacy endpoints of radiation therapy group protocol 0247: A randomized, phase 2 study of neoadjuvant radiation therapy plus concurrent capecitabine and irinotecan or capecitabine and oxaliplatin for patients with locally advanced rectal cancer. Int J Radiat Oncol Biol Phys 91:116-123, 2015 25446610 10.1016/j.ijrobp.2014.09.031 PMC4385459

[18] Palomaki GE Bradley LA Douglas MP, et al: Can UGT1A1 genotyping reduce morbidity and mortality in patients with metastatic colorectal cancer treated with irinotecan? An evidence-based review. Genet Med 11:21-34, 2009 19125129 10.1097/GIM.0b013e31818efd77 PMC2743611

[19] Shulman K Cohen I Barnett-Griness O, et al: Clinical implications of UGT1A1*28 genotype testing in colorectal cancer patients. Cancer 117:3156-3162, 2011 21287524 10.1002/cncr.25735 PMC3117027

[20] Innocenti F Undevia SD Iyer L, et al: Genetic variants in the UDP-glucuronosyltransferase 1A1 gene predict the risk of severe neutropenia of irinotecan. J Clin Oncol 22:1382-1388, 2004 15007088 10.1200/JCO.2004.07.173

[21] Rosner GL Panetta JC Innocenti F, et al: Pharmacogenetic pathway analysis of irinotecan. Clin Pharmacol Ther 84:393-402, 2008 18418374 10.1038/clpt.2008.63 PMC2759399

[22] Toffoli G Cecchin E Gasparini G, et al: Genotype-driven phase I study of irinotecan administered in combination with fluorouracil/leucovorin in patients with metastatic colorectal cancer. J Clin Oncol 28:866-871, 2010 20038727 10.1200/JCO.2009.23.6125 PMC4872310

[23] Marcuello E Páez D Paré L, et al: A genotype-directed phase I-IV dose-finding study of irinotecan in combination with fluorouracil/leucovorin as first-line treatment in advanced colorectal cancer. Br J Cancer 105:53-57, 2011 21654688 10.1038/bjc.2011.206 PMC3137420

[24] Toffoli G Sharma MR Marangon E, et al: Genotype-guided dosing study of FOLFIRI plus bevacizumab in patients with metastatic colorectal cancer. Clin Cancer Res 23:918-924, 2017 27507617 10.1158/1078-0432.CCR-16-1012 PMC6777349

[25] Innocenti F Schilsky RL Ramírez J, et al: Dose-finding and pharmacokinetic study to optimize the dosing of irinotecan according to the UGT1A1 genotype of patients with cancer. J Clin Oncol 32:2328-2334, 2014 24958824 10.1200/JCO.2014.55.2307 PMC4105486

[26] Zhu J Li X Shen Y, et al: Genotype-driven phase I study of weekly irinotecan in combination with capecitabine-based neoadjuvant chemoradiation for locally advanced rectal cancer. Radiother Oncol 129:143-148, 2018 29273261 10.1016/j.radonc.2017.11.026

[27] Quirke P Durdey P Dixon MF, et al: Local recurrence of rectal adenocarcinoma due to inadequate surgical resection. Histopathological study of lateral tumour spread and surgical excision. Lancet 2:996-999, 1986 2430152 10.1016/s0140-6736(86)92612-7

[28] Edge SB, Compton CC: The American Joint Committee on Cancer: the 7th edition of the AJCC cancer staging manual and the future of TNM. Ann Surg Oncol 17:1471-1474, 2010 10.1245/s10434-010-0985-4 20180029

[29] Rich TA Kirichenko AV: Camptothecin radiation sensitization: Mechanisms, schedules, and timing. Oncology (Williston Park) 12:114-120, 1998 (suppl 6) 9726103

[30] Klautke G Küchenmeister U Foitzik T, et al: Intensified irinotecan-based neoadjuvant chemoradiotherapy in rectal cancer: Four consecutive designed studies to minimize acute toxicity and to optimize efficacy measured by pathologic complete response. Radiother Oncol 85:379-384, 2007 18036687 10.1016/j.radonc.2007.10.042

[31] Kim KP Kim HS Sym SJ, et al: A UGT1A1*28 and *6 genotype-directed phase I dose-escalation trial of irinotecan with fixed-dose capecitabine in Korean patients with metastatic colorectal cancer. Cancer Chemother Pharmacol 71:1609-1617, 2013 23595344 10.1007/s00280-013-2161-6

[32] Liu CY Chen PM Chiou TJ, et al: UGT1A1*28 polymorphism predicts irinotecan-induced severe toxicities without affecting treatment outcome and survival in patients with metastatic colorectal carcinoma. Cancer 112:1932-1940, 2008 18300238 10.1002/cncr.23370

[33] Guan Y Shen Y Xu Y, et al: An expansion study of genotype-driven weekly irinotecan and capecitabine in combination with neoadjuvant radiotherapy for locally advanced rectal cancer with UGT1A1 *1*1 genotype. Therap Adv Gastroenterol 12:1756284819852293, 2019 10.1177/1756284819852293 PMC6557009 31217818

[34] Fokas E Allgäuer M Polat B, et al: Randomized phase II trial of chemoradiotherapy plus induction or consolidation chemotherapy as total neoadjuvant therapy for locally advanced rectal cancer: CAO/ARO/AIO-12. J Clin Oncol 37:3212-3222, 2019 31150315 10.1200/JCO.19.00308

[35] Zhu J Gu W Lian P, et al: A phase II trial of neoadjuvant IMRT-based chemoradiotherapy followed by one cycle of capecitabine for stage II/III rectal adenocarcinoma. Radiat Oncol 8:130, 2013 23718210 10.1186/1748-717X-8-130 PMC3680166

[36] Zhu J Liu F Gu W, et al: Concomitant boost IMRT-based neoadjuvant chemoradiotherapy for clinical stage II/III rectal adenocarcinoma: Results of a phase II study. Radiat Oncol 9:70, 2014 24606870 10.1186/1748-717X-9-70 PMC3984733

[37] Xu RH Muro K Morita S, et al: Modified XELIRI (capecitabine plus irinotecan) versus FOLFIRI (leucovorin, fluorouracil, and irinotecan), both either with or without bevacizumab, as second-line therapy for metastatic colorectal cancer (AXEPT): A multicentre, open-label, randomised, non-inferiority, phase 3 trial. Lancet Oncol 19:660-671, 2018 29555258 10.1016/S1470-2045(18)30140-2

[38] Innocenti F Vokes EE Ratain MJ: Irinogenetics: What is the right star? J Clin Oncol 24:2221-2224, 2006 16636339 10.1200/JCO.2005.05.2464

